# Venous thromboembolism risk stratification for patients with lower limb trauma and cast or brace immobilization

**DOI:** 10.1371/journal.pone.0217748

**Published:** 2019-06-20

**Authors:** D. Douillet, B. Nemeth, A. Penaloza, G. Le Gal, T. Moumneh, S. C. Cannegieter, P. M. Roy

**Affiliations:** 1 Department of Emergency Medicine, Angers University Hospital, MITOVASC Institute, University of Angers, Angers, France; 2 Department of Clinical Epidemiology, Leiden University Medical Center, Leiden, Netherlands; 3 Department of Orthopaedic Surgery, Leiden University Medical Center, Leiden, Netherlands; 4 Department of Emergency Medicine, Cliniques Universitaires Saint Luc, Université Catholique de Louvain, Brussels, Belgium; 5 Thrombosis Programme, Division of Hæmatology-Department of Medicine, University of Ottawa—Ottawa Hospital, Ottawa, Canada; 6 Department of Thrombosis and Hæmostasis, Leiden University Medical Center, Leiden, Netherlands; Monash University School of Public Health and Preventive Medicine, AUSTRALIA

## Abstract

**Background:**

Thromboprophylaxis for patients with non-surgical isolated lower-limb trauma requiring immobilization is a matter of debate. Our aim was to develop and validate a clinical risk- stratification model based on Trauma, Immobilization and Patients’ characteristics (the TIP score).

**Methods:**

The TIP score criteria and the cut-off were selected by a consensus of international experts (n = 27) using the Delphi method. Retrospective validation was performed in a population-based case-control study (MEGA study). The potential score’s impact in anticoagulant treatment was assessed in a prospective single-center observational cohort study.

**Findings:**

After four successive rounds, 30 items constituting the TIP score were selected: thirteen items for trauma, three for immobilization and 14 for patient characteristics were selected, each rated on a scale of 1 to 3. In the validation database, the TIP score had an AUC of 0·77 (95% CI 0.70 to 0.85). Using the cut-off proposed by the experts (≥5) and assuming a prevalence of 1·8%, the TIP scores had a sensitivity, specificity and negative predictive values of 89·9%, 30·7% and 99·4% respectively. In the prospective cohort, 84·2% (165/196) of all the patients concerned who presented at the emergency department had a low VTE risk not requiring thromboprophylaxis according to their TIP scores. The 3-month rate of symptomatic VTE was 1/196 [95% CI 0.1–2.8] this patient was in the sub-group TIP score ≥5.

**Conclusion:**

For patients with non-surgical lower-limb trauma and orthopedic immobilization, the TIP score allows an individual VTE risk-assessment and shows promising results in guiding thromboprophylaxis.

## Introduction

### Background

Isolated lower-limb trauma requiring cast immobilization is a common condition with several thousand patient admissions into emergency departments each day. Approximately 120,000 patients were admitted into US emergency departments for lower-limb injury in 2009 according to the National Electronic Injury Surveillance System (NEISS) [1]. Those patients are at risk of venous thromboembolism (VTE) owing to the venous stasis secondary to immobilization, hypercoagulability and vascular trauma, and they may be able to benefit from thromboprophylaxis [2–4]. In a recent case-control study, patients with a below-knee cast immobilization had an eight-fold increased risk of VTE within one year following cast application (OR 8·3 [95% CI 5·3 to 12·9]) [5]. However, the benefits of preventive anticoagulation remain unclear. A recent meta-analysis by the Cochrane library assessing low molecular weight heparin (LMWH) for VTE prophylaxis in patients with lower-limb cast immobilization included eight randomized controlled trials and showed that LMWH reduced the rate of VTE. However, the quality of evidence was moderate, especially due to the risks of selection and attrition biases. Moreover, low-quality trials were pooled with high-quality trials, thus diluting the effect of higher-quality studies. Therefore, the authors suggested that future research might give more directives on specific thromboprophylaxis advice for different types of patient or patient groups [6].

Trauma patients are heterogeneous and represent a wide range of VTE risk: some high-risk patients may benefit from anticoagulant treatment whereas, for others, this risk may be too low to justify thromboprophylaxis. Several research reports have shown that the VTE risk depends on the type of trauma (e.g. simple sprain vs severe fracture) as well as on the type of orthopedic immobilization (e.g. all lower-limb casting vs. below-knee brace) and on patient characteristics (e.g. young person with no medical history vs old person with a history of cancer and VTE), these different factors acting synergistically [5–8].

Our aim was to develop and validate a clinical risk-stratification model for patients with isolated non-surgical trauma of the lower limb requiring orthopedic immobilization in order to guide physicians for thromboprophylaxis treatment based upon individual risk-assessments.

### Methods

#### Design

**Fig 1:**
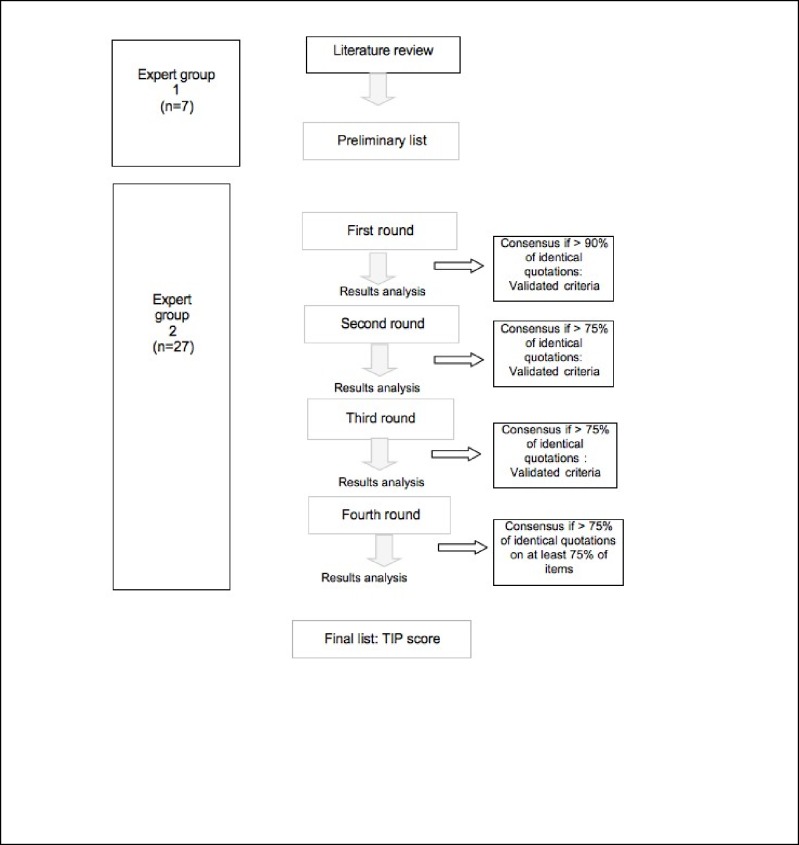
Flow-chart of the Delphi method.

We used the Delphi method to reach an expert consensus on VTE risk factors in patients with non-surgical lower-limb trauma requiring cast immobilization, and to perform a clinical decision-making model: the TIP (Trauma, Immobilization, Patients) score [9–10]. A validation of this score was performed in a large population-based case-control study: MEGA study (Fig 1) and the TIP score’s usability was assessed in a prospective cohort study.

#### Development of the TIP score using the Delphi method [9–10]

**Preliminary phase.** An initial list of potential VTE risk factors for patients with lower-limb trauma of the knee or below the knee and requiring immobilization was compiled by the study’s scientific committee. To this end, a comprehensive literature review was performed by the main investigators (DD and PMR). Publications were selected if they described VTE risk factors in our sub-group of interest and/or in the general population. Criteria were classified into three categories: criteria relating to trauma, immobilization or patient characteristics. A preliminary list of 110 potential risk factors (41 for the type of trauma, 16 for the type of immobilization and 53 for patient characteristics) was compiled. The list was notified to the members of the scientific committee, who were encouraged to retrieve or modify the proposed criteria and to add other potential VTE risk factors. The scientific committee comprised seven experts. Finally, the first list was made up of 76 items (S1 Table).

**Delphi panel.** The Delphi panel consisted of international multidisciplinary clinical experts (n = 27) whose eligibility was determined based upon their prior participation in collaborations and thromboembolic relevant publications indexed in PubMed/Medline. Unlike the principal investigator, members of the scientific committee were allowed to take part as experts. Anonymity of the panelists was assured throughout all Delphi rounds, i.e. the experts did not know who the other participants were, nor from whom answers, or commentaries had been obtained. With acceptance of the invitation, experts gave their informed consent to respect the rules of the Delphi Method, and to publication of the results (Table 1).

**Table 1:** Delphi experts’ characteristics.

Demographics	n = 27	%
**Gender**		
Male	24	89
Female	3	11
**State/territory**		
Belgium	3	11
Canada	2	7
France	12	44
Monaco	1	4
Netherlands	3	11
Spain	1	4
Switzerland	2	7
Tunisia	1	4
United States	2	7
**Expert category**		
Anaesthesiology	1	4
Cardiology	1	4
Emergency medicine	10	37
Internal medicine	2	7
Orthopaedic surgery	3	11
Pulmonologist	4	15
Vascular medicine	5	19
Vascular surgery	1	4

**Data collection.** Four rounds of expert consultations were performed between January and April 2017. The initial list of criteria was sent out to the experts as well as a list of references. Experts were asked to score each of the 76 items from 0 to 3. Zero was equivalent to "it is not a significant risk factor", 1 to "it is a low-risk factor", 2 to "it is an intermediate risk factor" and 3 to "it is a high-risk factor” for the onset of a thromboembolic event. The experts' comments and suggestions were relayed anonymously to the others at the next round. Criteria with an agreement between the experts >90% (absolute agreement) or >75% (strong agreement) were considered as validated. The others were subjected to a further round. From round to round, participants received the summary of results from the previous round and a questionnaire with an updated list of criteria. Questionnaires were sent out, and answers were collected electronically. The duration of each round was two weeks. To maximise participation, weekly e-mail reminders were sent to non-responders. On the final round, the experts were asked to group and simplify the criteria, which resulted in the final score named TIP score for trauma, immobilisation and patient. In addition, experts were asked to suggest a threshold value of VTE risk at which thromboprophylaxis should be administered.

**Statistical analysis.** Survey responses were summarized with descriptive statistics. Consensus (i.e., importance and agreement) was defined by examining the data distributions, mean and percentage of respondents rating. For the first round, criteria with a rate of agreement of over 90% were validated as absolute consensus. For the subsequent rounds, criteria with a rate of agreement of more than 75% were considered as a strong agreement. The study was considered positive if the agreement rate was greater than 75% for at least 75% of the items at the end of the fourth round. Data were analyzed using Microsoft Excel and the built-in tools from the SurveyMonkey website.

#### Validation of the TIP score

**Study design.** Retrospective validation was performed in the MEGA study (Multiple Environmental and Genetic Assessment of risk-factors for venous thrombosis). Details of this study have been published previously [11–13]. In short, 4,956 consecutive patients aged 18 to 70 and with a first deep-vein thrombosis (DVT), pulmonary embolism (PE), or both, were recruited from six anticoagulation clinics in Netherlands between 1 March 1999 and 31 August 2004. The diagnosis of DVT or PE was confirmed by (Doppler) ultrasonography, ventilation/perfusion scan, angiography, or a spiral CT scan. The control group (n = 6,297) consisted of partners of participating patients and other controls who were identified using a random digit dialling method; controls were frequency-matched to cases with respect to sex and age. All participants completed a questionnaire on VTE risk-factors such as trauma, immobilisation (including plaster-cast and cast location), (orthopaedic) surgery, current use of medication, if any, and comorbidity in the past year before the VTE event.

**Population.** For this analysis, cases and controls (n = 230, 194 cases and 36 controls) with a leg-cast in the MEGA study were used. After excluding those participants who also underwent surgery as part of their treatment, 176 cases and 33 controls were retained in the analysis.

**Statistical analysis.** Since for some patients, information on a few risk-factors was missing; we performed a multiple imputation technique to obtain complete data (10 imputations, results pooled in accordance with Rubin’s rules) [14]. Then the TIP score was calculated for each patient. Subsequently, the Area Under the Curve (AUC) was estimated by computing a Receiver Operating Characteristic. Sensitivity and specificity were calculated using the cut-off defined by the experts. Negative and positive predictive values were estimated assuming a prevalence of 1·8% [15].

We performed a sensitivity analysis, including only cases and controls in which the trauma component was known (n = 188, 163 cases, 25 controls). All validation analyses were performed in IBM SPSS Statistics for Windows, version 20·0 and Stata, version 12.

**Ethics.** Approval for this study was obtained from the Medical Ethics Committee of Leiden University’s Medical Centre, and all participants gave their written informed consent.

#### Usefulness assessment

**Study design**. In order to assess the proportion of patients for which thromboprophylaxis should be considered if the TIP score were applied using the cut-off defined by the experts, we performed a prospective single-centre observational cohort study. Our other objectives were to compare thromboprophylaxis with current practice, and to assess the 3-month rate of symptomatic VTE, all causes of deaths and bleeding.

**Population**. All patients with a non-surgical lower-limb trauma requiring an immobilization who gave their informed consent were included in the Emergency Department of Angers University Hospital. Clinical characteristics, including criteria of the TIP score and thromboprophylaxis decision in current practice, were collected prospectively. The TIP score was calculated retrospectively. Missing data were considered to be normal or absent. Patients were interviewed by telephone at the end of a 3-month follow-up period. End-points were the occurrence of a symptomatic VTE (distal or proximal deep vein thrombosis, pulmonary embolism or unexplained sudden deaths for which PE could not be excluded), bleeding or death. All possible events were externally adjudicated by an independent adjudication committee.

**Statistical analysis**. Data were summarized as means and standard deviations or as numbers and percentages, depending on the data type. Proportions are given with their 95% confidence interval (CI). Comparative analyses were performed using McNemar’s test, using p<0·05 for statistical significance.

**Ethics**. This study was approved by the Ethics Committee (ID-RCB: 2017-A00291-52) and declared on clinicaltrials.gov before inclusion of the first patient (NCT03089255).

## Results

### Development of the TIP score using the Delphi method

Four rounds were carried out, as defined *a priori*. The response-rate increased over successive rounds, 74% (20/27), 81% (22/27), 89% (24/27) and 93% (25/27), respectively. At the end of the rounds, all 76 criteria obtained a consensus considered at least as strong (>75%) (S2 Table). In the first round, two items obtained an absolute agreement (>90% identical answers) and were not submitted to the second round (n = 2/76; 2·6%). In the second round, 52 criteria were validated with an agreement rate >90% (n = 3/76; 3·9%) or >75% (n = 49/76; 64·5%). For the third round, 17 criteria reached a strong consensus >75% (n = 17/76; 22·4%). In the last round, a consensus was reached on the remaining five items (n = 5/76; 6·6%) (S2 Table).

The final score includes 30 criteria versus 76 on the first list. Eleven risk factors considered as not being clinically relevant were withdrawn: phalanx fracture(s), immobilization with plantar support, age less than 55 years, male sex, active smoking, known coronary artery disease, lower-limb arterial disease, liver failure, cirrhosis, diabetes, neuroleptic treatment. On the experts' proposal and in order to simplify the TIP score, 43 items were grouped together, resulting in 13 criteria (S2 Table). For example, BMI 25–35 kg/m^2^ and BMI >35 kg/m^2^ were consolidated into one criterion: BMI >30 kg/m^2^ scoring 1. Finally, four items were withdrawn. Known minor thrombophilia and other known hemostasis disorders were withdrawn because they were considered to be very rare in clinical practice in the absence of previous VTE. Quadriceps tendon rupture and distal femur fracture were left out because they were considered to require surgery most of the time, which did not correspond to the target population. For these regroupings or withdrawals, agreement rates ranged from 82% to 100% in the panel of experts.

The final TIP score includes 13 criteria for trauma, three criteria for immobilization, and 14 criteria for patient characteristics (Table 2). For trauma items, as for immobilization, a single item must be chosen (that which corresponds to the highest score) whereas for the characteristics of the patient, the scores of each item must be summed up. For example, a 62-year-old patient with a personal history of VTE and cancer requiring rigid immobilization below the knee owing to severe ankle sprain with forefoot dislocation will have a TIP score of 9 (T:2; I:2, P:3+1+1). The final score was approved by 25/26 experts (96%). One expert suggested reducing the number of risk-factors in order to improve the score’s clinical usability (S1 Table).

**Table 2:** Final list of TIP score.

Criteria of the Trauma Immobilisation Patient score		Score
*Only one item can be selected*		
**T**	Leg-bone fracture (tibia and fibula)	**3**
	Proximal tibia fracture	
	Ankle bi- or tri-malleolar fracture	**2**
	One leg-bone fracture (tibia or fibula)	
	Patellar fracture	
	Ankle or rear-foot dislocation	
	Severe ankle sprain (grade 3) or knee sprain (with severe oedema or haemarthrosis)	
	Achilles tendon rupture	
	Ankle isolated malleolar fracture	**1**
	Tarsal bone(s) or forefoot fracture	
	Proximal tibiofibular, patellar, mid-foot or forefoot dislocation	
	Moderate ankle sprain (grade 1 or 2) or knee sprain (without serious oedema or hæmarthrosis)	
	Major muscle injury	
*Only one item can be selected*		
I	Rigid immobilisation including the knee (resin or plaster)	**3**
	Rigid below-the-knee immobilisation (resin or plaster)	**2**
	Semi-rigid immobilisation without plantar support	**1**
*Multiple items can be selected*		
P	Known major thrombophilia* or personal history of VTE †	**3**
	Age >75 y‡	**2**
	Family history of VTE (first-degree relative)	**2**
	Active cancer or myelo-proliferative disorder	**2**
	Surgery within past 3 months	**2**
	Pregnancy and Puerperium (less than 6 months)	**2**
	Oestrogen hormone therapy (<2 y)	**2**
	Age >55 y and <75 y	**1**
	BMI >30kg/m^2^ §	**1**
	History of cancer	**1**
	Chronic venous insufficiency	**1**
	Bedridden within past 3 months or long travel/flight (>6 hours) or unilateral or bilateral lower-extremity paralysis	**1**
	Oestrogen hormone therapy (>2 y)	**1**
	Congestive heart failure NYHA >II ¶	**1**
	or chronic respiratory failure	
	or inflammatory bowel disease	
	or chronic kidney disease (GFR<50mL/min) ¥	

*Known major thrombophilia: antithrombin deficiency, homozygous factor V Leiden, homozygote mutation o the prothrombin gene, multiple thrombophilia.

† Personal history of VTE: DVT or PE.

‡ y: years

§ BMI: Body Mass Index

¶ NYHA: New York Heart Association's classification of cardiovascular diseases

¥ GFR: Glomerular filtration rate

The experts were asked to decide intuitively on a TIP score threshold value above which a thromboprophylaxis would be required. With a participation rate of 85% (23/27), results ranged from 3 to 9 with a median of 4, meaning that only patients with a TIP score greater than 4 (≥5) should be considered for thromboprophylaxis.

### Retrospective validation of the TIP score in the MEGA study

**Fig 2:**
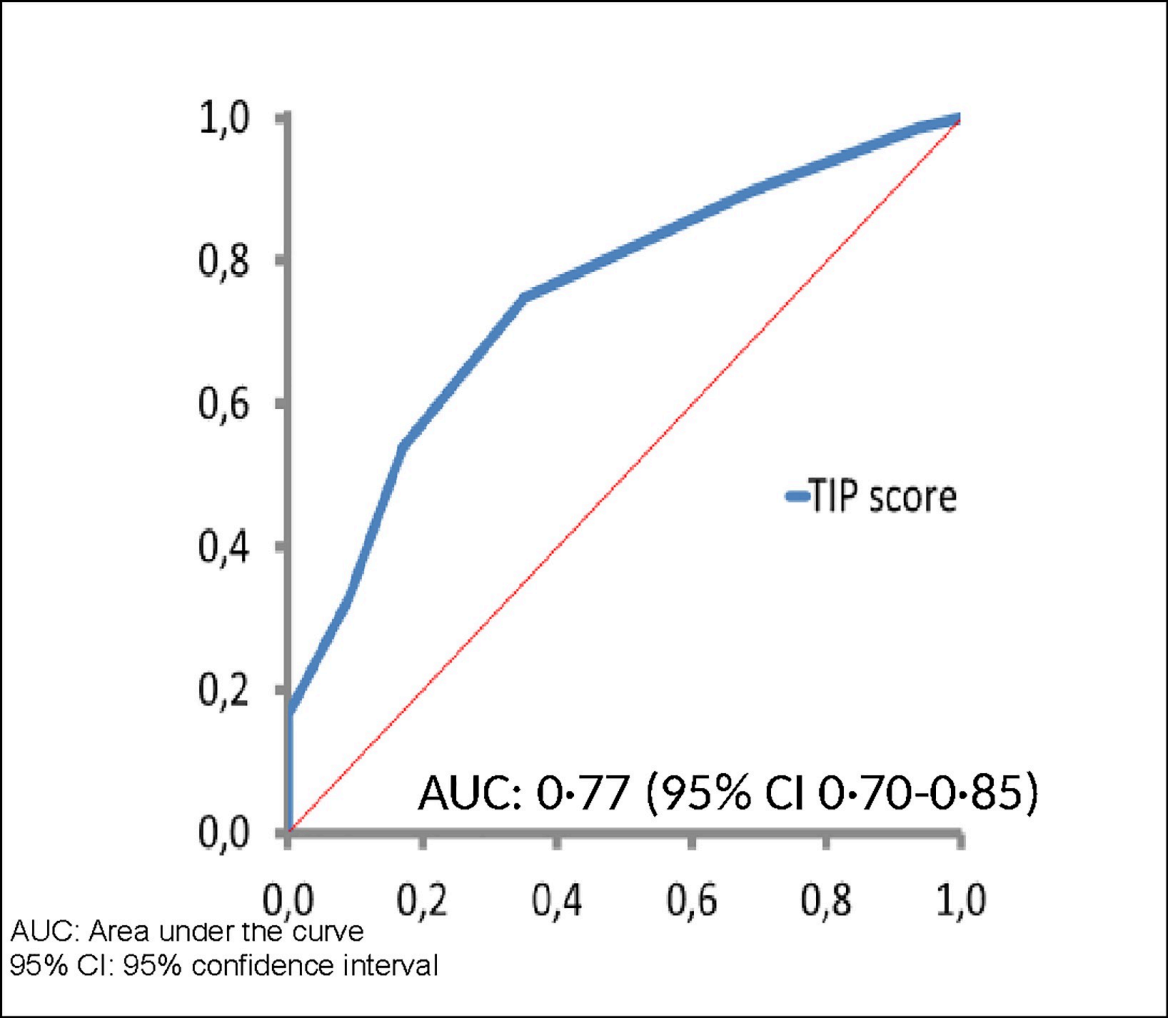
ROC curve derived from the MEGA study.

In the plaster-cast patients treated without surgery (n = 209; 176 patients and 33 control individuals), the TIP score ranged between 2 and 20 points (out of a maximum of 29 points for men and 35 for women). The TIP score had an AUC of 0.77 (95% CI 0.70 to 0.85) (Fig 2). Using 5 points as a cut-off (0–4: low-risk i.e. negative, ≥5: high-risk i.e. positive), the sensitivity was 89·9% while the specificity was 30·7% (Table 3). Assuming a prevalence of 1·8%, the negative predictive value was 99·4% and the positive predictive value 2·32%. Using the Youden index, the optimal threshold value was 6 points with 71·9%, 64·9%, 99·3% and 3·76% for sensitivity, specificity, negative and positive predictive values, respectively (Table 3).

**Table 3:** Predictive performance of the TIP score.

Cutpoint	Sensitivity	Specificity	PVV	NPV
**3**	100,0%	0,0%	1,80%	100,0%
**4**	98,5%	6,5%	1,89%	99,6%
**5**	89,9%	30,7%	2,32%	99,4%
**6**	74,9%	64,9%	3,76%	99,3%
**7**	53,9%	83,0%	5,49%	99,0%
**8**	32,8%	96,1%	13,31%	98,7%
**9**	16,6%	100,0%	100,00%	98,5%
**10**	7,3%	100,0%	100,00%	98,3%
**11**	3,2%	100,0%	100,00%	98,3%
**12**	0,8%	100,0%	100,00%	98,20%

In the sensitivity analysis including only patients with information about their type of trauma, 188 patients were included (163 cases and 25 control individuals). The TIP score had an AUC 0·77 (95% CI 0·69 to 0·85).

#### Usefulness assessment of the TIP score in the prospective study

Between May and September 2017, 197 consecutive patients with a non-surgical lower-limb trauma were included in the prospective study. One surgical patient was secondarily excluded. Baseline characteristics are summarized in Table 4. The TIP score was <5 for 165 of 196 patients (84·2% [95% CI 78·6 to 88·8) and ≥5 for 31 patients (15·8% [95% CI 11·2 to 21·4]). In accordance with standard practice, 72/196 (36·7%) patients received anticoagulant treatment for thromboprophylaxis, 52/165 (31·5%) among patients with a TIP score <5 and 20/31 (64·5%) among patients with a TIP score ≥5. If the TIP score had been applied, the anticoagulation rate would have been reduced by -20·9%, ([95% CI -15·7 to -27], p<0·05).

**Table 4:** Patients’ characteristics of the prospective cohort.

Patients	N = 196
Male sex __ no. (%) or means +/- SD	105 (53.3)
Age (years) __means ± SD	37.5±16.2
Body-mass index __ means ± SD*	25.7 ±6
Personal history of venous thromboembolism __ no. (%)	9 (4.6)
History of venous thromboembolism in first-degree relatives __ no. (%)†	16 (8.2)
Active cancer __ no. (%)‡	2 (1)
History of cancer __ no. (%)§	5 (2.6)
Surgery <3 months __ no. (%)	8 (4.1)
Recent bed rest __ no. (%)¶	6 (3.1)
Pregnancy __ no. (%)	2 (1)
Hormonal treatment __ no. (%)¥	29 (14.8)
Venous insufficiency __ no. (%)	10 (5.1)
**Trauma**	
Patellar fracture __ no./no. total (%)	1 (0.5)
Knee sprain with oedema / haemarthrosis__ no. (%)	7 (3.6)
Knee sprain without oedema / haemarthrosis__ no. (%)	11 (5.6)
Major muscle injury__ no./no. total (%)	3 (1.5)
Fracture of one leg bone (tibia or fibula) __ no. (%)	7 (3.6)
Ankle fracture: bi- and trimalleolar fracture__ no. (%)	9 (4.6)
Ankle fracture: isolated malleolar fracture__ no. (%)	2 (1)
Ankle sprain grade 3__ no. (%)	32 (16.3)
Ankle sprain grade 1 or 2 __ no. (%)	103 (52.6)
Achilles tendon rupture, non-surgical__ no. (%)	1 (0.5)
Fracture one (or more) tarsal bone(s) or forefoot__ no. (%)	20 (10.2)
Immobilisation	
Rigid including knee (resin or plaster) __ no. (%)	0 (0)
Rigid below the knee (resin or plaster) __ no. (%)	54 (27.6)
Semi-rigid without plantar support__ no. (%)	122 (62.2)
Other immobilisation__ no. (%)	20 (10.2)

* Missing value in 5 patients (N = 191).

† Missing value in 16 patients (N = 180).

‡ Active cancer is defined as cancer in the course of curative or palliative treatment, or known for less than 6 months.

§ History of cancer corresponds to non-active cancer.

¶ Recent bed rest over 3 days within past 3 months.

¥ Hormonal treatment is hormone replacement therapy or hormonal contraceptive, including oral contraceptives and intrauterine devices. Missing value in 16 patients (N = 180).

Six patients were lost to follow-up. One patient with a TIP score of = 5 did not receive thromboprophylaxis and developed proximal deep vein thrombosis one week after trauma and immobilization. The 3-month rate of symptomatic VTE was 0/160 (0%) [95% CI 0 to 2·3] in the sub-group of patients with a TIP score <5 and 1/30 (3·3%) [95% CI 0·6 to 16·7] in the sub-group of patients with a TIP score ≥5. No patient had major bleeding (0%, [95% CI 0·0 to 4·1%]), but three of 72 patients who received thromboprophylaxis had a non-major clinically relevant bleeding (4·2% [95% CI 1·4 to 11·6]).

## Discussion

Through a Delphi study involving an international multidisciplinary panel of experts and physicians, we classified thromboembolic risk-factors in patients with non-surgical lower-limb trauma requiring immobilization. A risk-stratification model based on trauma, immobilization and patient characteristics, i.e. the TIP score, was established. Validated retrospectively in a case-control study, the TIP score shows good prognostic performance (AUC 0·77). Using <5 as cut-off, the TIP score identified over 80% of patients as having a low risk of VTE, hence, no indication for thromboprophylaxis.

Current guidelines for thromboprophylaxis, and therefore also practices, differ widely among countries and centers, ranging from the absence of preventive anticoagulation to thromboprophylaxis for all patients for whom plantar support is not possible [16–19]. Both may be inappropriate. Indeed, recommendations for thromboprophylaxis are based mainly on small studies including heterogeneous and selected populations [20–24]. However, recently the largest multicenter randomized controlled trial performed thus far failed to demonstrate any beneficial effect of LMWH for VTE prevention in unselected patients with lower-limb casting [15]. Still, about 1·5%-2·0% of all patients develop VTE despite thromboprophylactic therapy [15]. Therefore, a new prophylactic strategy needs to be developed in order to prevent VTE in this large patient group. By using a risk-stratification model, low-risk patients can be withheld from thromboprophylaxis (and its downsides, i.e. bleeding, costs) whereas high-risk patients could benefit from treatment (i.e. prevent VTE), which should be of longer duration or higher dosage than the current strategy, as this is apparently not sufficient. For this purpose, the TIP score is a valuable and relevant decision-making aid.

Several methods can be used to develop a clinical decision-making aid model. In cases of heterogeneous and/or incomplete scientific data, the Delphi method is an appropriate and well-validated method [9–10]. It allows building a reliable model that is based upon scientific knowledge as well as clinical expertise. Our TIP score is the outcome of an international expert consensus that agreed on all the items presented through four successive rounds. With an AUC statistic of 0·77 (95% CI 0·70 to 0·85), the TIP score compares favorably with other risk-assessment models for VTE, such as the Qthrombosis for the general population (AUC of 0·75) [25], the Padua prediction score for hospitalized medical patients (AUC of 0·76) [26], or Trauma Embolic Scoring System (TESS) for severely injured patients (AUC of 0·71) [27]. Importantly, the TIP score appears to have at least similar performance to the L-TRiP (cast) score for patients with cast immobilisation developed from the MEGA-study [28]. Assessed, like the TIP score, in a sub-group of patients of the MEGA study with plaster-cast, the L-TRiP (cast) score has an AUC statistic of 0·76 (95% CI 0·66 to 0·86). Of note, many clinical variables of the L-TRIP (cast) score were incorporated by the experts into the TIP score. Nevertheless, the two scores have some relevant differences. For example, some items of the L-TRiP (cast) such as sex, pneumonia or superficial vein thrombosis were not considered by the experts as clinically relevant for decision-making at emergency departments. Conversely, the TIP score takes into account more variables, such as trauma and immobilisation characteristics, and apply to patients with semi-rigid immobilisation. Both the L-TRiP (cast) score and TIP score need prospective assessment and validation.

Our prospective observational study was the first stage of this process. Using the 5-point cut-off suggested by the expert, our results show that a large proportion of patients admitted into our emergency department for non-surgical lower-limb trauma requiring immobilization are classified as being a low-risk patient for VTE (<1%). Therefore, applying the TIP score could lead to a large decrease in the anticoagulation rate in some centers and countries. In a French national observational study, the overall rate of prophylactic treatment in non-surgical patients with lower-limb trauma and orthopedic immobilization was 61% [29]. Such a large decrease in LMWH prescription would reduce the discomfort and iatrogenic risk of daily injections: 1·6% of anticoagulated patients experienced clinically-significant bleeding in our study. On the other hand, the TIP score allowed identification of 16% of at-risk patients who might benefit from anticoagulation. Of note, 45% of high-risk patients according to the TIP score did not receive thromboprophylaxis in our single-center study. Moreover, high-risk patients might be candidates for other and possibly more powerful treatments than LMWH. Despite LMWH prevention, 1·4% of unselected patients with plaster-cast developed symptomatic VTE in the POT-CAST study [13]. Fondaparinux was more effective than nadroparin for preventing VTE after below-knee injury requiring prolonged immobilization in patients with additional risk-factors in a randomized controlled study [30], and a direct oral anticoagulant could be a valuable option for further investigation.

Nevertheless, our study does have some limitations. Firstly, although eligible experts for the Delphi method were carefully recruited, selection bias could not be excluded, many of them having already collaborated. Nevertheless, the panel included a heterogeneous group of researchers and clinicians from various countries and continents, and such heterogeneity strengthens the consensus statement and practical applicability worldwide. The experts defined the threshold value justifying thromboprophylaxis using their ‘gestalt’, not on the predictive performance of the TIP score. Nonetheless, this threshold appears to optimize the sensitivity of the score as comparing to the value obtained using the Youden index. Moreover, our findings demonstrate that multidisciplinary physician teams are able to agree on clinically detailed guidelines to make decisions on VTE risk-stratification. Our final score includes 30 criteria, which may be perceived as being a lot, a concern expressed by one expert. However, thanks to computerized clinical decision-support systems available on smartphones or other devices, this large number of criteria may not be disincentive. Indeed, such computerized decision-making aid systems have improved clinical practice at Emergency Departments [31]. Development of a computerized system for TIP scores is ongoing. Secondly, the MEGA study database contains a large number of patients. However, after selecting our population of interest the validation population remains modest (230 patients, 194 cases and 36 controls). Some data were missing, especially regarding trauma characteristics. Nevertheless, our sensitivity analysis confirmed that the results were similar both with and without imputation for missing data. Since the MEGA study is a case-control study, we had to apply a predefined prevalence (1·8% on the basis of the POT-CAST study) in order to calculate the predictive values of the TIP score. Finally, our observational prospective study was single-centre and not empowered to demonstrate the safety of TIP score implementation. Nevertheless, our results are encouraging and support further assessments.

In conclusion, for patients with non-surgical lower-limb trauma and orthopedic immobilization, the TIP score, based on an international experts’ consensus using the Delphi method, allows an individual VTE risk-assessment and shows promising results in terms of its safety and usefulness for guiding thromboprophylaxis. An implementation validation study is now required.

## Supporting information

S1 Table Initial list of criteria selected following literature analysis by the first expert group. (DOC)

S2 Table Final list of TIP score with the rounds of the consensus and consensus agreement level. (DOC)

## References

[1] Lambers K, Ootes D, Ring D. Incidence of patients with lower extremity injuries presenting to US emergency departments by anatomic region, disease category, and age. Clin Orthop Relat Res. 1 2012;470(1):284–90. 10.1007/s11999-011-1982-z 21785896 PMC3237997

[2] Kujath P, Spannagel U, Habscheid W. Incidence and prophylaxis of deep venous thrombosis in outpatients with injury of the lower limb. Haemostasis. 3 1993;23 Suppl 1:20–6. 8388353 10.1159/000216905

[3] Knudson MM, Morabito D, Paiement GD, Shackleford S. Use of low molecular weight heparin in preventing thromboembolism in trauma patients. J Trauma. 9 1996;41(3):446–59. 8810961 10.1097/00005373-199609000-00010

[4] Kudsk KA, Fabian TC, Baum S, Gold RE, Mangiante E, Voeller G. Silent deep vein thrombosis in immobilized multiple trauma patients. Am J Surg. 12 1989;158(6):515–9. 2589580 10.1016/0002-9610(89)90182-7

[5] van Adrichem RA, Debeij J, Nelissen RGHH, Schipper IB, Rosendaal FR, Cannegieter SC. Below-knee cast immobilization and the risk of venous thrombosis: results from a large population-based case-control study. J Thromb Haemost. 9 2014;12(9):1461–9. 10.1111/jth.12655 25040873

[6] Zee AAG, van Lieshout K, van der Heide M, Jansseen L, Janzing HMJ. Low molecular weight heparin for prevention of venous thromboembolism in patients with lower-limb immobilization. Cochrane Database of Systematic Reviews 2017, Issue 8 Art. No.: CD006681 10.1002/14651858.CD006681.pub4 28780771 PMC6483324

[7] van Stralen KJ, Rosendaal FR, Doggen CJM. Minor injuries as a risk factor for venous thrombosis. Arch Intern Med. 14 1 2008;168(1):21–6. 10.1001/archinternmed.2007.5 18195191

[8] Rosendaal FR. Venous thrombosis: a multicausal disease. Lancet. 3 4 1999;353(9159):1167–73. 10.1016/s0140-6736(98)10266-0 10209995

[9] Black N, Murphy M, Lamping D, McKee M, Sanderson C, Askham J, et al Consensus development methods: a review of best practice in creating clinical guidelines. J Health Serv Res Policy. 10 1999;4(4):236–48. 10.1177/135581969900400410 10623041

[10] Diamond IR, Grant RC, Feldman BM, Pencharz PB, Ling SC, Moore AM, et al Defining consensus: a systematic review recommends methodologic criteria for reporting of Delphi studies. J Clin Epidemiol. 4 2014;67(4):401–9. 10.1016/j.jclinepi.2013.12.002 24581294

[11] Blom JW, Doggen CJM, Osanto S, Rosendaal FR. Malignancies, prothrombotic mutations, and the risk of venous thrombosis. JAMA. 9 2 2005;293(6):715–22. 10.1001/jama.293.6.715 15701913

[12] Bezemer ID, Doggen CJM, Vos HL, Rosendaal FR. No association between the common MTHFR 677C->T polymorphism and venous thrombosis: results from the MEGA study. Arch Intern Med. 12 3 2007;167(5):497–501. 17353498 10.1001/archinte.167.5.497

[13] Cannegieter SC, Doggen CJM, van Houwelingen HC, Rosendaal FR. Travel-related venous thrombosis: results from a large population-based case control study (MEGA study). PLoS Med. 8 2006;3(8):e307 10.1371/journal.pmed.0030307 16933962 PMC1551914

[14] Marshall A, Altman DG, Holder RL, Royston P. Combining estimates of interest in prognostic modelling studies after multiple imputation: current practice and guidelines. BMC Med Res Methodol. 2009;9:57 10.1186/1471-2288-9-57 19638200 PMC2727536

[15] van Adrichem RA, Nemeth B, Algra A, le Cessie S, Rosendaal FR, Schipper IB, et al Thromboprophylaxis after Knee Arthroscopy and Lower-Leg Casting. New England Journal of Medicine. 9 2 2017;376(6):515–25. 10.1056/NEJMoa1613303 27959702

[16] Falck-Ytter Y, Francis CW, Johanson NA, Curley C, Dahl OE, Schulman S, et al Prevention of VTE in orthopedic surgery patients: Antithrombotic Therapy and Prevention of Thrombosis, 9th ed: American College of Chest Physicians Evidence-Based Clinical Practice Guidelines. Chest. 2 2012;141(2 Suppl):e278S–e325S. 22315265 10.1378/chest.11-2404 PMC3278063

[17] French Society of Anaesthesia and Intensive Care. Guidelines on perioperative venous thromboembolism prophylaxis. Update 2011. Short text. Ann Fr Anesth Reanim. 2011 12;30(12):947–51 [erratum in: 2012 Jan;31(1):93]. 10.1016/j.annfar.2011.10.008 22104443

[18] National Institute for Clinical Excellence. Venous thromboembolism: reducing the risk. Reducing the risk of venous thromboembolism (deep vein thrombosis and pulmonary embolism) in patients admitted to hospital. NICE guideline 92. National Institute for Clinical Excellence, 2010:50.

[19] Geerts WH, Pineo GF, Heit JA, Bergqvist D, Lassen MR, Colwell CW, et al Prevention of venous thromboembolism: the Seventh ACCP Conference on Antithrombotic and Thrombolytic Therapy. Chest. 9 2004;126(3 Suppl):338S–400S. 15383478 10.1378/chest.126.3_suppl.338S

[20] Lapidus LJ, Ponzer S, Elvin A, Levander C, Lärfars G, Rosfors S, et al Prolonged thromboprophylaxis with Dalteparin during immobilization after ankle fracture surgery: a randomized placebo-controlled, double-blind study. Acta Orthop. 8 2007;78(4):528–35. 10.1080/17453670710014185 17966008

[21] Lassen MR, Borris LC, Nakov RL. Use of the low-molecular-weight heparin reviparin to prevent deep-vein thrombosis after leg injury requiring immobilization. N Engl J Med. 5 9 2002;347(10):726–30. 10.1056/NEJMoa011327 12213943

[22] Jørgensen PS, Warming T, Hansen K, Paltved C, Vibeke Berg H, Jensen R, et al Low molecular weight heparin (Innohep) as thromboprophylaxis in outpatients with a plaster cast: a venografic controlled study. Thromb Res. 15 3 2002;105(6):477–80. 12091045 10.1016/s0049-3848(02)00059-2

[23] Testroote M, Stigter W, de Visser DC, Janzing H. Low molecular weight heparin for prevention of venous thromboembolism in patients with lower-leg immobilization. Cochrane Database Syst Rev. 8 10 2008;(4):CD006681 10.1002/14651858.CD006681.pub2 18843725

[24] Dalkey N, Helmer O. An Experimental Application of the DELPHI Method to the Use of Experts. Management Science. 1 4 1963;9(3):458–67.

[25] Hippisley-Cox J, Coupland C. Development and validation of risk prediction algorithm (QThrombosis) to estimate future risk of venous thromboembolism: prospective cohort study. BMJ. 16 8 2011;343:d4656 10.1136/bmj.d4656 21846713 PMC3156826

[26] Barbar S, Noventa F, Rossetto V, Ferrari A, Brandolin B, Perlati M, et al A risk assessment model for the identification of hospitalized medical patients at risk for venous thromboembolism: the Padua Prediction Score. J Thromb Haemost. 11 2010;8(11):2450–7. 10.1111/j.1538-7836.2010.04044.x 20738765

[27] Ho KM, Rao S, Rittenhouse KJ, Rogers FB. Use of the Trauma Embolic Scoring System (TESS) to predict symptomatic deep vein thrombosis and fatal and non-fatal pulmonary embolism in severely injured patients. Anaesth Intensive Care. 11 2014;42(6):709–14. 10.1177/0310057X1404200605 25342402

[28] Nemeth B, Adrichem RAv, Hylckama Vlieg Av, Bucciarelli P, Martinelli I, Baglin T. Venous Thrombosis Risk after Cast Immobilization of the Lower Extremity: Derivation and Validation of a Clinical Prediction Score, L-TRiP(cast), in Three Population-Based Case–Control Studies. Sattar N, editor. PLoS Med. 2015 11 10; 12(11):e1001899 10.1371/journal.pmed.1001899 26554832 PMC4640574

[29] Riou B, Rothmann C, Lecoules N, Bouvat E, Bosson J-L, Ravaud P, et al Incidence and risk factors for venous thromboembolism in patients with nonsurgical isolated lower limb injuries. Am J Emerg Med. 6 2007;25(5):502–8. 10.1016/j.ajem.2006.09.012 17543652

[30] Samama C-M, Gafsou B, Jeandel T, Laporte S, Steib A, Marret E, et al [French Society of Anaesthesia and Intensive Care. Guidelines on perioperative venous thromboembolism prophylaxis. Update 2011. Short text]. Ann Fr Anesth Reanim. 12 2011;30(12):947–51. 10.1016/j.annfar.2011.10.008 22104443

[31] Kawamoto K, Houlihan CA, Balas EA, Lobach DF. Improving clinical practice using clinical decision support systems: a systematic review of trials to identify features critical to success. BMJ. 2 4 2005;330(7494):765 10.1136/bmj.38398.500764.8F 15767266 PMC555881

