# Supplementary material for: Venous thromboembolism risk stratification for patients with lower limb trauma and cast or brace immobilization
Source: PLoS One. 2019 Jun 20;14(6):e0217748. doi: 10.1371/journal.pone.0217748 (PMC6586277; doi:10.1371/journal.pone.0217748)
Supplement: S1 Table — (DOC) [file pone.0217748.s001.doc]

**S1 Table.** Initial list of criteria selected following literature analysis by the first expert group (L1).

| Trauma  ▪ Thigh and knee  Serious muscle injury  Quadriceps tendon rupture  Patellar luxation  Patellar fracture  Non-severe knee sprain (without oedema / hæmarthrosis)  Severe knee sprain (with oedema or/and hæmarthrosis)  Distal femur fracture (supra- and/or intercondylar fracture, non-displaced, etc.)  Proximal tibia fracture (tibia plateau fracture, tibia eminence fracture, etc.)  ▪ Leg, ankle, rear foot  Serious muscle injury  Isolated diaphysis tibia fracture  Isolated diaphysis fibular fracture  Fracture of leg bones  Proximal tibiofibular dislocation  Single malleolar ankle fracture  bi or tri-malleolar ankle fracture  Ankle or rear-foot dislocation  Ankle sprain grade 1  Ankle sprain grade 2  Severe ankle sprain grade 3  Achilles tendon rupture  ▪ Mid-foot and forefoot  Mid-foot or forefoot dislocation  Tarsal bone or forefoot fracture  Phalangeal fracture  Immobilisation  ▪ With plantar support  Flexible knee brace  Flexible ankle brace  Knee brace, type Zimmer  Knee splint orthopaedic immobilisation/articulated  Semi-rigid splint  Forefoot relief shoes  Posterior ankle splint  ▪ With plantar support  Crutches without immobilisation  Flexible knee brace  Flexible ankle brace  Knee brace, type Zimmer  Knee splint orthopaedic immobilisation/articulated  Semi-rigid splint  Forefoot relief shoes  Posterior ankle splint  Complete long leg cast (resin or plaster)  Leg cylinder cast (resin or plaster)  Short leg cast (resin or plaster)  Characteristics of patient  Age <35y  Age >55y and <75y  Age >75y  Male sex  Personal history of VTE provoked  Personal history of VTE unprovoked  Family history of VTE (firstdegree relative)  BMI >25 and <35Kg/m²  BMI >35Kg/m²  Active smoking  Active cancer (metastasis and/or having received chemotherapy or radiotherapy within past 6 months)  History of cancer  Myelo-proliferative disorders  Surgery within past 3 months  Unilateral or bilateral lower-extremity paralysis  Pregnancy  Puerperium (less than 6 months)  Oestrogenic hormone therapy (contraception / replacement therapy) under way for less than two years  Oestrogenic hormone therapy (contraception / replacement therapy) under way for more than two years  Travel with flight >6 hours  Congestive heart failure NYHA >II  Coronary artery disease  Arteriosclerosis obliterans of the lower limbs  Chronic respiratory failure  Inflammatory bowel disease  Nephrotic syndrome  Chronic kidney disease (GFR <50ml/min)  Chronic liver failure (INR >1.5)  Cirrhosis  Diabetes  Chronic venous insufficiency  Psychiatric disorder under neuroleptic therapy  ▪ Biological factors  Known major thrombophilia (Antiphospholipid syndrome, protein S deficiency, protein C deficiency, Antithrombin deficiency,  homozygous or doubly heterozygous for factor V of Leiden and the prothrombin gene)  Known minor thrombophilia (heterozygous for factor V of Leiden and the prothrombin gene, …)  Other known haematosis disorders (non O blood type, white-blood-cell count > 10G/L, haematocrit >50%). |
| --- |
