# Supplementary material for: Venous thromboembolism risk stratification for patients with lower limb trauma and cast or brace immobilization
Source: PLoS One. 2019 Jun 20;14(6):e0217748. doi: 10.1371/journal.pone.0217748 (PMC6586277; doi:10.1371/journal.pone.0217748)
Supplement: S2 Table — (DOC) [file pone.0217748.s002.doc]

**S2 Table.** Final list of TIP score with the rounds of the consensus and consensus agreement level.

| **Criteria of the TIP score** | Number of turns to reach consensus | Agreement rate | Score |
| --- | --- | --- | --- |
| **TRAUMA (only one can be chosen)** | | | |
| Fracture of leg bones  Proximal tibia fracture | 3  2 | >75%  >75% | **+3** |
| Ankle fracture : bi- and tri-malleolar fracture  *Fracture of a long leg bone (tibia or fibula)*  Patellar fracture  Ankle or rear-foot dislocation  *Ankle sprain grade 3 / Severe or knee with oedema / haemarthrosis*  Achilles tendon rupture | 2  2-4  3  2  4-3  2 | >75%  >75%  >75%  >75%  >75%  >75% | **+2** |
| Ankle fracture: Isolated malleolar fracture  Fracture one (or more) tarsal bone(s) or forefoot  *Proximal tibio-fibular dislocation or Patellar or mid-foot*  *or forefoot*  *Simple knee sprain without oedema / haemarthrosis or*  *ankle (grade 1 or 2)*  *Major muscle injury* | 3  2  2-2-2  2-2-2  2-2 | >75%  >75%  >75%  >75%  >75% | **+1** |
| **IMMOBILISATION** | | | |
| *Rigid including knee (resin or plaster)* | 2-2 | >75% | **+3** |
| Rigid below the knee (resin or plaster) | 3 | >75% | **+2** |
| *Semi-rigid without support* | 2-4 | >75% | **+1** |
| **PATIENT** | | | |
| Known major thrombophilia  or *Personal history of VTE (provoked - unprovoked)* | 1  3-2 | > 90%  > 90% | **+3** |
| Age >75 years  Family history of VTE (first-degree relative)  *Active cancer or Myelo-proliferative disorders*  Surgery within past 3 months.  *Pregnancy and Puerperium (less than 6 months)*  Oestrogenic hormone therapy (<2 years) | 2  2  2-2  3  2-2  2 | >75%  >75%  >75%  >75%  >75%  >75% | **+2**  **+2**  **+2**  **+2**  **+2**  **+2** |
| Age >55 years and <75 years  BMI >30kg/m2*  History of cancer  Chronic venous insufficiency  *Bedridden within the past 3 months.*  *or Travel with flight >6 hours   or Unilateral or bilateral lower-extremity paralysis*  Oestrogenic hormone therapy (>2 years)  *Congestive heart failure NYHA >II   or Chronic respiratory failure  or Inflammatory bowel diseases  or Chronic kidney disease (GFR<50mL/min)* | 3  -  2  2  3  2  3  2  2  4  3  2 | >75%  100 %  >75%  > 90%  >75%  >75%  >75%  >75%  >75%  > 90%  >75%  > 90% | **+1**  **+1**  **+1**  **+1**  **+1**  **+1**  **+1** |

*In italics: associated items defined in the fourth round.*

*: Threshold value redefined at the fourth round.
